# An evaluation of the comparative effectiveness of geriatrician-led comprehensive geriatric assessment for improving patient and healthcare system outcomes for older adults: a protocol for a systematic review and network meta-analysis

**DOI:** 10.1186/s13643-017-0460-4

**Published:** 2017-03-24

**Authors:** Charlene Soobiah, Caitlin Daly, Erik Blondal, Joycelyne Ewusie, Joanne Ho, Meghan J. Elliott, Rossini Yue, Jayna Holroyd-Leduc, Barbara Liu, Sharon Marr, Jenny Basran, Andrea C. Tricco, Jemila Hamid, Sharon E. Straus

**Affiliations:** 1 Li Ka Shing Knowledge Institute, St. Michael’s Hospital, 209 Victoria Street, East Building, Room 716, Toronto, Ontario M5B 1T8 Canada; 2 Institute of Health Policy Management and Evaluation, University of Toronto, 155 College Street Suite 425, Toronto, Ontario M5T 3M6 Canada; 3 Department of Health Research Methods, Evidence, and Impact, McMaster University, 1280 Main Street West, Hamilton, Ontario L8S 4K1 Canada; 4 Schlegel Research Institute for Aging, 250 Laurelwood Drive, Waterloo, Ontario N2J 0E2 Canada; 5 Department of Medicine, University of Calgary, Foothills Hospital, 11th Floor South Tower, Room 1103, 1403-29 Street NW, Calgary, Alberta T2N 2T9 Canada; 6 Regional Geriatrics Program of Toronto, Sunnybrook Health Sciences Centre, 2075 Bayview Avenue, L-wing, 1st floor, room L1-01D, Toronto, Ontario M4N 3M5 Canada; 7 Regional Geriatrics Program Central, McMaster University, 88 Maplewood, Hamilton, Ontario L8M 1W9 Canada; 8 Department of Medicine, University of Saskatchewan, 701 Queen St, Saskatoon, Saskatchewan S7K 0M7 Canada; 9 Dalla Lana School of Public Health, University of Toronto, 155 College Street 6th floor, Toronto, Ontario M5T 3M6 Canada; 10 Department of Medicine, University of Toronto, 200 Elizabeth Street, Suite R. Fraser Elliott 3-805, Toronto, Ontario M5G 2C4 Canada

**Keywords:** Systematic review, Network meta-analysis, Comprehensive geriatric assessment, Geriatric care, Geriatric assessment, Integrated knowledge translation

## Abstract

**Background:**

Comprehensive geriatric assessment (CGA) is an integrated model of care involving a geriatrician and an interdisciplinary team and can prioritize and manage complex health needs of older adults with multimorbidity. CGAs differ across healthcare settings, ranging from shared care conducted in primary care settings to specialized inpatient units in acute care. Models of care involving geriatricians vary across healthcare settings, and it is unclear which CGA model is most effective. Our objective is to conduct a systematic review and network meta-analysis (NMA) to examine the comparative effectiveness of various geriatrician-led CGAs and to identify which models improve patient and healthcare system level outcomes.

**Methods:**

An integrated knowledge translation approach will be used and knowledge users (KUs) including patients, caregivers, geriatricians, and healthcare policymakers will be involved throughout the review. Electronic databases including MEDLINE, EMBASE, Cochrane library, and Ageline will be searched from inception to November 2016 to identify relevant studies. Randomized controlled trials of older adults (≥65 years of age) that examine geriatrician-led CGAs compared to any intervention will be included. Primary and secondary outcomes will be selected by KUs to ensure the results are relevant to their decision-making. Two reviewers will independently screen the search results, extract data, and assess risk of bias. Data will be synthesized using an NMA to allow for multiple comparisons using direct (head-to-head) as well as indirect evidence. Interventions will be ranked according to their effectiveness using surface under the cumulative ranking curve (SUCRA).

**Discussion:**

As the proportion of older adults grows worldwide, the demand for specialized geriatric services that help manage complex health needs of older adults with multimorbidity will increase in many countries. Results from this systematic review and NMA will enhance decision-making and the efficient allocation of scarce geriatric resources. Moreover, active involvement of KUs throughout the review process will ensure the results are relevant to different levels of decision-making.

**Systematic review registration:**

PROSPERO CRD42014014008

**Electronic supplementary material:**

The online version of this article (doi:10.1186/s13643-017-0460-4) contains supplementary material, which is available to authorized users.

## Background

Caring for older adults (≥65 years) with complex health and functional needs remains a challenge in healthcare. Approximately 67% of older adults have ≥2 chronic conditions and often receive care from several clinicians and take multiple medications [1–3]. As a result, they often experience fragmented care and are exposed to drug-disease, drug-drug, and disease-disease interactions, which may lead to reduced function and quality of life [2]. Integrated models of care such as comprehensive geriatric assessments (CGAs) can prioritize and address the complex health needs of older adults with multimorbidity. CGAs focus on diagnosing and managing medical, psychological, functional, and social capabilities of older adults and involve collaboration with a geriatrician and an interdisciplinary team [4]. CGAs occur in different models of care across healthcare settings, ranging from shared care in primary care settings to specialized inpatient units in acute care settings. Given the variation of CGAs across the healthcare continuum, it is unclear which models are most effective to meet the needs of older adults with multimorbidity.

Several systematic reviews suggest that CGAs can improve patient outcomes. A Cochrane review of CGAs identified 22 randomized trials; compared to usual care, those who received a CGA in acute care settings were more likely to remain in their home, have improved cognition and a lower risk of mortality [4]. In outpatient settings, CGAs reduced functional decline and admissions to nursing homes when compared to usual care [5]. Similar results were also noted in geriatrician-led CGAs in rehabilitation settings when compared to usual care [6].

While these systematic reviews suggest that CGAs can impact patient and healthcare system outcomes, it is unclear which model of CGA is optimal or what the role of the geriatrician should be in the respective models. Several systematic reviews considered interdisciplinary teams or unit-specific assessment (i.e., geriatric evaluation and management units) for CGA but did not separate geriatrician-led CGAs from nurse or primary care physician-led CGAs [5, 7–10]. One systematic review examined geriatrician-led CGAs in rehabilitation settings and found a beneficial effect; however, it is unclear whether geriatrician-led CGAs in other settings (i.e., acute care, long-term care) are also effective [6].

Understanding optimal roles for geriatricians across healthcare settings is crucial, given the limited number of geriatricians in Canada. Geriatricians belong to a subspecialty of internal medicine and integrate care for older adults across multiple and often complex comorbidities, such as geriatric syndromes, and frailty [11]. In 2015, there were approximately 261 practicing geriatricians in Canada, which translates to less than one geriatrician per 100,000 Canadians [12]. The ratio of geriatricians is unlikely to increase significantly to meet the demands of the aging population, as there were only 21 trainees in 2016 pursuing this subspecialty [13]. As a result, we are faced with a situation where the demand for specialized geriatric care outweighs the number of specialized physicians and the demand will only escalate in the next few years. Therefore, it is important to understand which geriatrician-led models of care are effective, so we can target scarce resources effectively and make optimal use of geriatricians’ knowledge and skills.

To address this gap, we plan to conduct a systematic review and network meta-analysis (NMA) to determine the comparative effectiveness of geriatrician-led CGAs across healthcare settings. An integrated knowledge translation (iKT) approach will be used and knowledge users (KUs), such as patients, caregivers, geriatricians, and policymakers, will be engaged throughout. Our research question is: For adults aged ≥65 years, what is the comparative effectiveness of geriatrician-led models of care for improving patient-level (e.g., function, quality of life, caregiver stress) and healthcare system-level (e.g., admission to long-term care) outcomes?

## Methods

The systematic review and NMA will be conducted using methods established by the Cochrane Collaboration [14]. To facilitate uptake and to ensure results are applicable to multiple levels of decision-making, an iKT approach will be used to engage KUs in the review process. To guide KU engagement, the Stakeholder Engagement in Comparative Effectiveness Research (SECER) Framework will be used [15, 16]. The SECER framework postulates that KUs will be more engaged in research when results are deliberated, contextualized, and aligned with their values and preferences, the full framework is presented in Additional file 1 [16]. To accomplish this, we have embedded several qualitative components within the systematic review process to seek KU preferences and values. Our systematic review will be conducted in several stages.

## Part 1—Systematic review protocol

The protocol was drafted according to the PRISMA protocol statement [17], and revised with feedback from geriatricians, a systematic review methodologist, and a statistician. The final protocol was registered with PROSPERO (CRD42014014008) [18].

### Eligibility criteria

Randomized controlled trials (RCTs) that include adults aged ≥65 years from all healthcare settings (e.g., primary care, acute care, long-term care, and rehabilitation) are eligible. RCTs were selected, as they are the most validated study design for evaluating the effectiveness of healthcare interventions [19, 20]. The intervention must focus on a geriatrician-led care model (e.g., solo member, team-based interventions) across the healthcare continuum, such as clinics (e.g., shared care with primary care), home visits, inpatient consultation, inpatient and outpatient rehabilitation, day hospital, telehealth, and acute inpatient units. Care must focus on CGA, including assessment and management of medical, psychological, functional, and social capability. Standard care or other care models (geriatrician-led or not) will be considered as comparators. Primary and secondary outcomes will be selected by KUs to ensure the review is relevant for their decision making. Potential patient outcomes may include cognition, function (basic and instrumental activities of daily living), quality of life, caregiver stress, and patient satisfaction. Potential healthcare system level outcomes may include: number of patients living at home (inverse of death or living in residential care [4]), number of emergency department visits, number of acute care admissions, admission to long-term care, death, length of stay in acute care, number of outpatient visits, healthcare utilization, and number of physician visits. No restrictions will be placed on the year of publication, language, or publication status. Eligibility criteria are presented in Additional file 2.

### Information sources and literature search

A comprehensive literature search will be conducted by an experienced librarian (Ms. Becky Skidmore) in consultation with the research team. Electronic databases including MEDLINE, EMBASE, Cochrane Library, and Ageline will be searched from inception to November 2016, using keywords such as “geriatric assessment”, “health services for the aged”, and “comprehensive health care”. A draft search strategy is presented in Additional file 3. The literature search will be peer reviewed by another information specialist (Ms. Heather MacDonald) using the PRESS checklist and modified accordingly [21]. To supplement our electronic search strategy, we will search clinical trial databases such as BioMed Central ISRCTN registry or the National Institutes of Health clinical trial registry, as well as conference proceedings from relevant organizations such as the Canadian Geriatric Society, Canadian Association of Gerontology, and the International Association of Gerontology and Geriatrics to identify unpublished trials. In addition, we will scan the reference lists of included studies to obtain RCTs not captured by our literature search.

### Study selection process

The search results will be uploaded to *Synthesi.SR* [22], a proprietary systematic review software owned by the Knowledge Translation Program at St. Michael’s Hospital. It allows screening of citations and full-text articles by multiple reviewers simultaneously. Prior to reviewing search results, a pilot test of 150 citations will be conducted to ensure reliability amongst reviewers. Inter-rater reliability will be assessed using percent agreement and >80% will be indicative of good reliability amongst reviewers. Two reviewers will subsequently screen titles/abstracts and full-text articles for inclusion independently. Discrepancies will be mediated by a third reviewer.

### Charting

After relevant studies are identified, the outcomes and outcome measures reported in CGA studies will be charted in Excel. A description of variables captured in the charting file is presented in Additional file 4. The purpose of charting is to identify which outcome measures are reported in the literature so that KUs can select the outcomes that are most relevant for their decision-making. Additional PICO elements (i.e., the length of study, the number of time points, comorbid conditions) will be abstracted but will only be used to inform the full data abstract form and potential statistical analyses.

## Part 2—Selection of outcomes and measures for inclusion in systematic review

### Knowledge users

Patients, caregivers, geriatricians, and policymakers will be invited to participate as KUs. Patients and caregivers will be recruited from the Elders’ Clinic at St. Michael’s Hospital and will be approached if they have had a CGA in the last year and do not have a diagnosis of dementia. Geriatricians will be recruited through the Division of Geriatric Medicine, University of Toronto, and policymakers or healthcare managers will be recruited from the Regional Geriatrics Program of Ontario, Health Quality Ontario and decision makers associated with the Council of Academic Hospitals of Ontario. We hope to engage 30 KUs in the systematic review process. Ethics approval was obtained from St. Michael’s Hospital and the University of Toronto.

### Delphi process to select relevant outcomes

A modified Delphi approach with two rounds will be used to achieve consensus on which outcomes should be considered for inclusion [23]. First, an online survey will be sent to KUs (via *Qualtrics* [24]) with a list of all outcomes reported in the CGA literature. Additional considerations will be made for patients and caregivers, as they may not have email access, or feel comfortable using the online platform. A research assistant will be available to help patients and caregivers with the survey either by phone or in person. KUs will rate the importance of each outcome on a 7-point Likert scale ranging from “not at all important” to “extremely important”. Ratings will be aggregated and median ratings along with interquartile ranges will be calculated. Outcomes with median ratings of ≥5 will be considered important outcomes for decision-making and move on to the second round. In the second round, KUs will rate the shortened list of preferred outcomes using the same scale. The two-step elimination approach will ensure that only the outcomes that are most relevant to their decision-making needs are included in the systematic review.

### Survey to select appropriate outcome measures

After outcomes are selected, an online cross-sectional survey with geriatricians will be conducted to identify optimal validated measures for each outcome. Patients, caregivers, and policymakers typically do not use these measures and will not participate. Only continuous patient-level outcomes (i.e., cognition, function, quality of life) will be included in the survey, as there are many ways these outcomes can be measured. For example, in a previous charting exercise of Alzheimer’s dementia, >70 outcome measures were identified in the literature for assessing cognition [25]. It is not feasible to abstract data on all of these measures and similarly, this is not practical for NMA. As such, we feel this additional step will streamline data abstraction for continuous outcomes and reduce outcome heterogeneity in statistical analyses. Geriatricians will rate the outcome measures using the same approach that was used for selecting outcomes, with a 7-point Likert ranging from “not at all important” to “extremely important”. Validated outcome measures with median ratings of ≥5 will be considered for inclusion in the systematic review, increasing the relevance of results.

## Part 3—Completion of the systematic review

### Data abstraction

A standardized data abstraction form will be created and tailored to outcome measures selected in part 2. The form will include study characteristics (e.g., study design, year trial conducted, sample size, setting, country of trial conduct, intervention, and comparator details), patient characteristics (e.g., type and number of patients, mean age and standard deviation, co-morbidities), intervention characteristics (e.g., role of geriatrician on team, members of the care team, care setting), and outcomes selected by KUs (e.g., number of events per treatment arm, means, standard deviation).

The data abstraction form will be piloted with the review team using a random sample of 10 included RCTs and modified as needed. Data abstraction will begin when sufficient agreement is noted (i.e., percent agreement >80%). To ensure accuracy, two reviewers will independently abstract all data using the standardized form in Excel; discrepancies will be resolved by a third reviewer, who will verify all data. In some studies, outcome results will be reported over many time periods but only the longest duration will be abstracted [14]. Multiple publications may report data from the same study group (i.e., companion reports). When this occurs, data will only be abstracted for the longest duration if different from the original study to avoid duplicate publication bias. Information captured from companion reports will be used for supplementary data only. Authors will be contacted to obtain missing data such as details of the study setting, or intervention (e.g., the role of geriatrician, frequency of assessments).

### Methodological quality and risk of bias assessment

Risk of bias will be assessed using the Cochrane Risk of Bias Tool [26]. The tool considers the internal validity of included RCTs and focuses on random sequence, allocation concealment, blinding of participants and personnel, incomplete outcome data, selective reporting, and other sources of bias (i.e., funding bias). To assess the extent of publication bias, comparison-adjusted funnel plots will be used when more than 10 studies are available [27]. Comparison-adjusted funnel plots are an extension of funnel plots and account for multiple pair-wise comparisons [27].

### Data synthesis

The results from the systematic review will be summarized descriptively and study characteristics, patient characteristics, risk of bias results, and frequencies of outcomes across the included RCTs will be reported.

### Meta-analysis

A Bayesian meta-analysis (MA) will be conducted to examine pairwise comparisons. Bayesian MA offers some advantages over traditional frequentist methods as parameter uncertainty is automatically accounted for in the analysis and a Bayesian approach can facilitate probabilistic statements which can enhance decision making [28]. In addition, the approach allows for direct comparison between mixed treatment comparisons in the NMA and direct head-to-head results from MA [28]. MA will be conducted whenever two or more studies compare the same two interventions and comparators for the same outcome. We expect that study and patient characteristics will differ across trials and we will apply a random effects model to incorporate anticipated heterogeneity. Statistical heterogeneity will be evaluated using the *I* ^2^ statistic. If significant statistical heterogeneity is observed (e.g., *I* ^2^ > 75%), a meta-regression and/or subgroup analysis will be conducted [29]. The meta-regression will explore the influence baseline effect sizes; age, frailty, comorbidities, cognition, function, setting (all sources of clinical heterogeneity); and risk of bias results (source of methodological heterogeneity) on MA results. The number of covariates examined will be constrained to one tenth of the number of studies to avoid type II errors [30]. If a meta-regression is not possible (<10 studies), subgroup analyses will be used to explore the sources of clinical and methodological heterogeneity on results. Potential subgroups that may be explored include care setting (primary versus other), the role of the geriatrician (shared care versus most responsible physician), risk of bias (high versus low), and attrition rate (<10 versus ≥10%). Some RCTs will not report relevant data (e.g., standard deviations) needed for analysis, and to include these in the analysis, missing continuous data will be imputed using established methods [31].

The Bayesian MA will be conducted in OpenBUGS [32]. Results will be reported as odds ratios for dichotomous outcomes or mean difference for continuous outcomes, along with 95% credible intervals based on 100,000 Markov chain Monte Carlo simulations after a burn in of at least 50,000 simulations and vague priors. The aim of the vague prior is to elicit comparable results that would have been obtained through frequentist inference; however, results may be sensitive to vague priors [33]. To examine this influence of priors on point estimates, sensitivity analyses will be conducted using different priors. Model convergence will be assessed using the trace and history plot functions in OpenBUGS, as well as the Brooks-Gelman-Rubin (BGR) statistic [34]. Forest plots will be generated using the metafor package in R statistical program [35].

### Network meta-analysis

If appropriate, a Bayesian NMA will be conducted to synthesize results for each outcome. Nodes in the NMAs will represent the various geriatrician-led care models. CGAs are complex interventions that are often tailored to individual needs and implemented in different healthcare settings. We will define nodes to lessen the potential heterogeneity in the network. The role of the geriatrician and the healthcare setting of the CGA are known factors that contribute to the diversity of CGAs. Geriatricians will be consulted during node categorization to ensure that similar geriatrician roles are grouped together, which will aid in the clinical interpretation of results. Depending on the breadth of included studies and the extent of heterogeneity examined by simple classification, we may also consider dividing the nodes further into components and examine the members of the multidisciplinary teams and the frequency of contact with the multidisciplinary team and geriatrician.

For NMA results to be valid, assumptions of comparability, such as connectivity, transitivity, and consistency need to be considered [36]. Connectivity refers to how well the studies form a connected network and will be visualized using network diagrams [37]. Although there is no formal definition of what constitutes a connected network, the following will be used as a guide; a connected network should link the different interventions from each study and enable the connection of at least two interventions in the evidence base, and for every two studies, there should be at least one common comparator. Sparse or unconnected networks are not suitable for NMA, due to the heavy reliance on indirect evidence and will be assessed on their network geometry [38]. Once connectivity is observed, transitivity will be explored, which allows us to make indirect comparisons by way of a common comparator (i.e., standard care) [36]. To assess whether indirect comparisons are valid, we will color the edges in the NMA diagram to represent patient and study characteristics and assess visually whether there is an equal distribution of these characteristics across the interventions [36]. Patient and study characteristics that we anticipate may influence transitivity include frailty, comorbid conditions, gender, and healthcare setting (i.e., acute care, long-term care settings). The consistency of the results from direct evidence (i.e., head-to-head trials from pairwise MA) versus indirect evidence (i.e., from NMA) will be compared using the design-by-treatment model [39], which assesses global inconsistency across the network. If global inconsistency is observed, we will check for data abstraction errors and if none exist, we will explore local inconsistency using the loop-specific method [40]. If inconsistency is still observed, we will conduct meta-regression and/or subgroup analysis. Potential meta-regression or subgroup analyses that we may explore will include age, frailty, cognition, functional status, attrition (<10 versus ≥10%), care setting (acute care, primary care, long-term care settings), and risk of bias (high versus low).

All NMAs will be conducted in OpenBUGS [32] Bayesian statistical software. Results will be reported as odds ratios for dichotomous outcomes or mean difference for continuous outcomes, along with 95% credible intervals based on 100,000 Markov chain Monte Carlo simulations after a burn in of at least 50,000 simulations and vague priors. Model convergence will be assessed using the trace and history plot functions in OpenBUGS, as well as the Brooks-Gelman-Rubin (BGR) statistic [34]. Predictive intervals will be calculated, which gives an indication of how the results will change if a new trial is conducted in the future and added to the evidence. The effectiveness of care models will be ranked using surface under the cumulative ranking (SUCRA) curve [37]. Sensitivity analysis will be conducted to examine the effects of imputations for missing data [31].

### Dissemination

After analysis, KUs will participate in a 1-day meeting to discuss the results. A nominal group technique will be used to develop the key messages for each KU group (i.e., patients/caregivers, clinicians, and policymakers) [41]. Specifically, all NMA results will be presented to KUs at the meeting and they will work in small groups to create the key messages and develop the dissemination strategy for each audience. Key messages will be highlighted in the final systematic review manuscript.

At the end of the 1-day meeting, KUs will complete the patient and public engagement questionnaire (PPEQ), which is an online survey to quantify engagement [42]. It uses criteria from the SECER framework and is a process measure that provides a qualitative indication of how engaged KUs feel in the research process [16]. Use of the PPEQ is novel to systematic reviews but has the potential to provide empirical evidence for the impact of the iKT process. Use of the questionnaire will allow us to understand if surveys, nominal group technique, or Delphi’s are effective methods to engage stakeholders in the systematic review process.

The dissemination strategy will be informed by the Knowledge-to-Action framework [43]. A spectrum of end-of-grant knowledge translation strategies will be explored and will be tailored to different audiences. Our strategy will include passive dissemination via publication in peer-reviewed journals, the creation of knowledge tools (such as slide decks for our KUs to use in their dissemination activities as knowledge brokers), and activities such as providing interactive workshops and videos. Our approach is as follows: the design of key messages will be clear, simple, action-oriented, and tailored for each KU audience; sources of the message will be individuals that are influential and credible with each target audience (i.e., KUs as knowledge brokers); and dissemination approaches will be KU-driven and tailored to how and when they want to receive information.

## Discussion

Geriatrician-led CGAs have the potential to help older adults manage complex healthcare needs; however, their effectiveness is unclear. Several systematic reviews have examined CGAs across healthcare settings; however, it is still unclear which model is most effective or what the role of the geriatrician should be in the respective models. There is a lack of head-to-head trials in the CGA literature and our systematic review can overcome some of the limitations of previous reviews and meta-analyses through the use of NMA. NMA explores comparative effectiveness by using the totality of information available and compares interventions that have not been assessed in head-to-head studies. Using direct and indirect evidence in an NMA can increase power and precision of estimators. In addition, the approach allows us to rank the interventions in terms of probability of being the most effective or safest, which can facilitate informed decision-making.

The iKT approach will actively engage KUs in the research process, by selecting relevant outcomes and measures for inclusion in the review and analysis. In addition, KUs will identify the key messages resulting from the review and the information can be used to inform implementation and dissemination strategies, such as patient decision aids or clinical practice guidelines, which will have a direct impact on the delivery of healthcare. Additionally, this will help refine knowledge products and help to further increase the uptake of research findings stemming from the systematic review and NMA.

## Limitations and challenges

The current study is a large and comprehensive review of CGAs and there are several limitations and challenges to our methods. First, in the data abstraction phase of our systematic review, we are only abstracting data on the longest duration of follow-up, which may inadvertently contribute to a lack of consistency across studies and result in heterogeneity in MA or NMA applications. However, if there is a robust amount of studies reporting multiple time points we may consider conducting several analyses stratified by length of duration. In addition, we will not be able to explore intervention effects over time (e.g., 3 months versus 6 months). We anticipate some degree of inconsistency and have taken steps to limit undue heterogeneity in data, such as selecting appropriate outcomes measures which may reduce outcome heterogeneity. We feel this approach is conservative and will make data abstraction streamlined and will enable us to conduct an NMA.

Second, our NMA considers the assumptions underlying the statistical approach; however, the data may violate these assumptions and it may not be feasible or appropriate to conduct an NMA. If an NMA is not possible, we will report on the synthesis results for all pairwise comparisons using Bayesian MA. The MA will provide an update of results from previous systematic reviews and we will examine differences in point estimates from previous analyses. Connectivity and transitivity assumptions are contingent on the similarity of interventions within nodes. Our nodes represent the role of the geriatrician in the respective models of care and may not be explicitly stated in the trials; as such, it may be difficult to categorize nodes and will make the synthesis of evidence a challenge. To overcome this, we will consult with geriatricians during the process of node categorization to increase the chances of creating clinically relevant nodes.

Lastly, we plan on recruiting 30 knowledge users (KUs) to be part of the systematic review process. KUs will not screen search results, abstract data, or assist with critical appraisal. KUs will provide input along the way and will contribute to the overall direction of the systematic review. This may limit their engagement in the process as they will not be involved in all steps of the systematic review. We felt this approach was most feasible, as it did not require any training or require major time commitments and is an adequate level of engagement that will be useful to the review and KUs.

## Additional files

Additional file 1: Stakeholder Engagement in Comparative Effectiveness Research (SECER) Framework. Conceptual framework which will guide conduct of knowledge user engagement in the systematic review. (DOCX 28 kb)

Additional file 2: Eligibility criteria. List of criteria for selection of relevant studies. (DOCX 14 kb)

Additional file 3: Preliminary MEDLINE Literature Search. Search strategy that will be used to identify citations and will be modified for the respective databases. (DOCX 14 kb)

Additional file 4: Charting exercise. Description of variable that will be captured from relevant citations. (DOCX 19 kb)

Additional file 5: Authorization for funding. Funding agreement with the Canadian Institutes of Health Research (CIHR) and the Strategy for Patient Oriented Research (SPOR). (PDF 45 kb)

## Abbreviations

CGA: Comprehensive geriatric assessment
iKT: Integrated knowledge translation
KU: Knowledge user
MA: Meta-analysis
NMA: Network meta-analysis
RCT: Randomized controlled trial
SECER: Stakeholder Engagement in Comparative Effectiveness Research

## References

[1] Terner M, Reason B, McKeag AM, Tipper B, Webster G (2011). Chronic conditions more than age drive health system use in Canadian seniors. Healthc Q.

[2] Boyd CM, McNabney MK, Brandt N (2012). Guiding principles for the care of older adults with multimorbidity: an approach for clinicians: American Geriatrics Society Expert Panel on the Care of Older Adults with Multimorbidity. J Am Geriatr Soc.

[3] Salive ME (2013). Multimorbidity in older adults. Epidemiol Rev.

[4] Ellis G, Whitehead MA, O’Neill D, Langhorne P, Robinson D. Comprehensive geriatric assessment for older adults admitted to hospital. Cochrane Database Syst Rev. 2011;(7):CD006211. doi:10.1002/14651858.CD006211.pub2. 10.1002/14651858.CD006211.pub2 PMC4164377 21735403

[5] Stuck AE, Egger M, Hammer A, Minder CE, Beck JC (2002). Home visits to prevent nursing home admission and functional decline in elderly people: systematic review and meta-regression analysis. JAMA.

[6] Bachmann S, Finger C, Huss A, Egger M, Stuck AE, Clough-Gorr KM (2010). Inpatient rehabilitation specifically designed for geriatric patients: systematic review and meta-analysis of randomised controlled trials. BMJ.

[7] Ellis G, Whitehead MA, Robinson D, O’Neill D, Langhorne P (2011). Comprehensive geriatric assessment for older adults admitted to hospital: meta-analysis of randomised controlled trials. BMJ.

[8] Van Craen K, Braes T, Wellens N (2010). The effectiveness of inpatient geriatric evaluation and management units: a systematic review and meta-analysis. J Am Geriatr Soc.

[9] Huss A, Stuck AE, Rubenstein LZ, Egger M, Clough-Gorr KM (2008). Multidimensional preventive home visit programs for community-dwelling older adults: a systematic review and meta-analysis of randomized controlled trials. J Gerontol A Biol Sci Med Sci.

[10] Conroy SP, Stevens T, Parker SG, Gladman JR (2011). A systematic review of comprehensive geriatric assessment to improve outcomes for frail older people being rapidly discharged from acute hospital: ‘interface geriatrics’. Age Ageing.

[11] Heckman GA, Molnar FJ, Lee L (2013). Geriatric medicine leadership of health care transformation: to be or not to be?. Can Geriatr J.

[12] Canadian Medical Association. Geriatr Med Profile. 2015. https://www.cma.ca/Assets/assets-library/document/en/advocacy/Geriatric-e.pdf. Accessed 15 Dec 2016.

[13] Service CRM (2016). Medical subspecialty match—number of matched applicants by first choice discipline and quotas.

[14] Higgins J, Green S (2011). Cochrane handbook for systematic reviews of interventions version 5.1.0. The Cochrane collaboration.

[15] Deverka PA, Lavallee DC, Desai PJ (2012). Facilitating comparative effectiveness research in cancer genomics: evaluating stakeholder perceptions of the engagement process. J Comp Eff Res.

[16] Deverka PA, Lavallee DC, Desai PJ (2012). Stakeholder participation in comparative effectiveness research: defining a framework for effective engagement. J Comp Eff Res.

[17] Moher D, Shamseer L, Clarke M (2015). Preferred reporting items for systematic review and meta-analysis protocols (PRISMA-P) 2015 statement. Syst Rev.

[18] University of York. PROSPERO: International prospective register of systematic reviews. 2015. www.crd.york.ac.uk/PROSPERO/. Accessed 23 Oct 2016.

[19] Laupacis A, Straus S (2007). Systematic reviews: time to address clinical and policy relevance as well as methodological rigor. Ann Intern Med.

[20] Petticrew M, Roberts H (2003). Evidence, hierarchies, and typologies: horses for courses. J Epidemiol Community Health.

[21] McGowan J, Sampson M, Salzwedel DM, Cogo E, Foerster V, Lefebvre C (2016). PRESS Peer Review of Electronic Search Strategies: 2015 Guideline Statement. J Clin Epidemiol.

[22] Synthesi.sr. 2014. http://breakthroughkt.ca/login.php. Accessed 23 Oct 2016.

[23] Dalkey N, Helmer O (1963). An experimental application of the Delphi method to the use of experts. Manag Sci.

[24] Qualtrics - Qualitative Survey software. 2016. https://www.qualtrics.com. Accessed 03 Dec 2016.

[25] Soobiah C, Straus SE, Blondal E, Ghassemi M, Khan PA, Ho J, Berliner S, Tricco AC (2012). Outcomes utilized to assess Alzheimer’s disease from a systematic review and survey of clinicians and decision-makers.

[26] Higgins JP, Altman DG, Gotzsche PC (2011). The Cochrane Collaboration’s tool for assessing risk of bias in randomised trials. BMJ.

[27] Veroniki AAH-MTB, Fountoulakis KN, Biondi-Zoccai G (2016). Appraising between-study homogeneity, small study effects, moderators, and confounders. Umbrella reviews—evidence synthesis with overviews of reviews and meta-epidemiologic studies.

[28] Sutton AJ, Abrams KR (2001). Bayesian methods in meta-analysis and evidence synthesis. Stat Methods Med Res.

[29] Higgins JP, Thompson SG (2002). Quantifying heterogeneity in a meta-analysis. Stat Med.

[30] Peduzzi P, Concato J, Kemper E, Holford TR, Feinstein AR (1996). A simulation study of the number of events per variable in logistic regression analysis. J Clin Epidemiol.

[31] Carpenter J, Rucker G, Schwarzer G (2011). Assessing the sensitivity of meta-analysis to selection bias: a multiple imputation approach. Biometrics.

[32] The bugs project: WinBUGS. 2015. https://www.mrc-bsu.cam.ac.uk/software/bugs/. Accessed 23 Oct 2016.

[33] Lambert PC, Sutton AJ, Burton PR, Abrams KR, Jones DR (2005). How vague is vague? A simulation study of the impact of the use of vague prior distributions in MCMC using WinBUGS. Stat Med.

[34] Gelman A, Rubin DB. Inference from iterative simulation using multiple sequences. Stat Sci. 1992:457-472.

[35] R Project for Statistical Computing .2015. http://www.r-project.org. Accessed 23 Oct 2016.

[36] Jansen JP, Trikalinos T, Cappelleri JC (2014). Indirect treatment comparison/network meta-analysis study questionnaire to assess relevance and credibility to inform health care decision making: an ISPOR-AMCP-NPC Good Practice Task Force report. Value Health.

[37] Salanti G, Ades AE, Ioannidis JP (2011). Graphical methods and numerical summaries for presenting results from multiple-treatment meta-analysis: an overview and tutorial. J Clin Epidemiol.

[38] Salanti G, Kavvoura FK, Ioannidis JP (2008). Exploring the geometry of treatment networks. Ann Int Med.

[39] Veroniki AA, Vasiliadis HS, Higgins JP, Salanti G (2013). Evaluation of inconsistency in networks of interventions. Int J Epidemiol.

[40] Dias S, Welton NJ, Caldwell DM, Ades AE (2010). Checking consistency in mixed treatment comparison meta-analysis. Stat Med.

[41] Andersen B, Fagerhaug T (2000). The nominal group technique: generating possible causes and reaching consensus. Qual Prog.

[42] Moore A, Brouwers M, Straus SE, Tonelli M (2015). Advancing patient and public involvement in guideline development.

[43] Graham ID, Logan J, Harrison MB (2006). Lost in knowledge translation: time for a map?. J Contin Educ Health Prof.

