# Supplementary material for: An evaluation of the comparative effectiveness of geriatrician-led comprehensive geriatric assessment for improving patient and healthcare system outcomes for older adults: a protocol for a systematic review and network meta-analysis
Source: Syst Rev. 2017 Mar 24;6:65. doi: 10.1186/s13643-017-0460-4 (PMC5366126; doi:10.1186/s13643-017-0460-4)
Supplement: Supplementary file 1 — Stakeholder Engagement in Comparative Effectiveness Research (SECER) Framework. Conceptual framework which will guide conduct of knowledge user engagement in the systematic review. (DOCX 28 kb) [file 13643_2017_460_MOESM1_ESM.docx]

**Additional file 1. Stakeholder Engagement in Comparative Effectiveness Research (SECER) Framework**

**INPUTS**

- Stakeholder preferences, values, and experiences in different models of CGA
- Information derived from the literature

**Types of Evidence**

**METHODS**

**OUTPUTS**

**Quantitative**

- Delphi exercise (Part 2)
- Cross sectional survey (Part 2)

**Qualitative**

- Nominal Group technique (Part 3)
- Selection of outcomes for inclusion in systematic review (Part 2)
- Selection of outcome measures for synthesis (Part 2)
- Prioritization of research findings (Phase 3)

**Process:** Patient and public engagement questionnaire (PPEQ)

**Decisions**

**Comparative Effectiveness Research:** Network meta-analysis (Part 2)

**Methods for combining experience and evidence**

**Outcomes**

**Adapted from**: Deverka PA, et al. Stakeholder participation in comparative effectiveness research: defining a framework for effective engagement*. J Comp Eff Res*. 2013; 1(2): 181-194
