# Supplementary material for: An evaluation of the comparative effectiveness of geriatrician-led comprehensive geriatric assessment for improving patient and healthcare system outcomes for older adults: a protocol for a systematic review and network meta-analysis
Source: Syst Rev. 2017 Mar 24;6:65. doi: 10.1186/s13643-017-0460-4 (PMC5366126; doi:10.1186/s13643-017-0460-4)
Supplement: Supplementary file 2 — Eligibility criteria. List of criteria for selection of relevant studies. (DOCX 14 kb) [file 13643_2017_460_MOESM2_ESM.docx]

**Additional file 2. Eligibility Criteria**

1. Does this study include older adults (≥65 years old)?
   1. YES
   2. NO
   3. UNSURE
2. Is this study a randomised controlled trial?
   1. YES
   2. NO
   3. UNSURE
3. Does this study include a comprehensive geriatric assessment?
   1. YES
   2. NO
   3. UNSURE
4. Does this study include a geriatrician?
   1. YES
   2. NO
   3. UNSURE
5. Is there a suitable comparator? (i.e., usual care or another geriatrician-led model of care)
   1. YES
   2. NO
   3. UNSURE
