# Supplementary material for: An evaluation of the comparative effectiveness of geriatrician-led comprehensive geriatric assessment for improving patient and healthcare system outcomes for older adults: a protocol for a systematic review and network meta-analysis
Source: Syst Rev. 2017 Mar 24;6:65. doi: 10.1186/s13643-017-0460-4 (PMC5366126; doi:10.1186/s13643-017-0460-4)
Supplement: Supplementary file 3 — Preliminary MEDLINE Literature Search. Search strategy that will be used to identify citations and will be modified for the respective databases. (DOCX 14 kb) [file 13643_2017_460_MOESM3_ESM.docx]

**Additional file 3. Preliminary MEDLINE Literature search**

Database: Ovid MEDLINE(R) In-Process & Other Non-Indexed Citations and Ovid MEDLINE(R) <1946 to Present> Search Strategy:

--------------------------------------------------------------------------------

1 Health Services for the Aged/

2 ((geriatric* or psychogeriatric* or psycho-geriatric* or gerontolog* or elder* or older adult* or older person* or older people or older population* or old-age or senior*) adj5 (care or daycare or healthcare or health service$1 or homecare or home nurs* or home support* or house call* or framework* or mobile health or model$1 or rehabilit* or remote consultat* or specialist* or speciali#ed or telehealth or tele-health or telemedicine or tele-medicine or telenurs* or tele-nurs* or unit or units or ward or wards)).tw.

3 (GEMU or GEMUs).tw.

4 Geriatric Assessment/

5 ((geriatric* or psychogeriatric* or psycho-geriatric* or gerontolog* or alzheimer* or dement*) adj5 (assess* or consult* or diagnos* or exam or exams or examin* or evaluat*)).tw.

6 CGA.tw.

7 ((geriatric* or psychogeriatric* or psycho-geriatric* or gerontolog* or alzheimer* or dement* or elder* or older adult* or older person* or older people or older population* or old-age) adj5 (collaborat* or comanag* or co-manag* or coordinat* or co-ordinat* or comprehensive or multidisciplinary or multi-disciplinary or multidimension* or multi-dimension* or integrat* or interconnect* or inter-connect* or interdisciplinary or inter-disciplinary or interprofessional* or inter-professional*)).tw.

8 or/1-7

9 Geriatrics/

10 (geriatric* or psychogeriatric* or psycho-geriatric* or gerontolog*).tw.

11 9 or 10

12 exp Patient Care/

13 exp Patient Care Team/

14 exp Community Health Services/

15 exp Comprehensive Health Care/

16 exp Delivery of Health Care/

17 Diagnostic Services/

18 exp Hospital Units/

19 exp Mental Health Services/

20 Models, Organizational/

21 exp Nursing Homes/

22 Inpatients/

23 Outpatients/

24 exp Rehabilitation/

25 exp Telemedicine/

26 or/12-25

27 11 and 26

28 8 or 27

29 (controlled clinical trial or randomized controlled trial).pt.

30 clinical trials as topic.sh.

31 (randomi#ed or randomly or RCT$1 or placebo*).tw.

32 ((singl* or doubl* or trebl* or tripl*) adj (mask* or blind* or dumm*)).tw.

33 trial.ti.

34 or/29-33

35 28 and 34

36 exp Animals/ not (exp Animals/ and Humans/)

37 35 not 36

38 (comment or editorial or interview or letter or news).pt.

39 37 not 38
