# Supplementary material for: An evaluation of the comparative effectiveness of geriatrician-led comprehensive geriatric assessment for improving patient and healthcare system outcomes for older adults: a protocol for a systematic review and network meta-analysis
Source: Syst Rev. 2017 Mar 24;6:65. doi: 10.1186/s13643-017-0460-4 (PMC5366126; doi:10.1186/s13643-017-0460-4)
Supplement: Supplementary file 4 — Charting exercise. Description of variable that will be captured from relevant citations. (DOCX 19 kb) [file 13643_2017_460_MOESM4_ESM.docx]

**Additional file 4. Charting Exercise**

| **Variable** | **Definition** | **Details** |
| --- | --- | --- |
| **Study Identification** | To identify where the study came from | DROP DOWN MENU (Initial search, Scanning reference list, Unpublished data ) |
| **REFID** | Reference identification | Text box |
| **Author** | First author name | Text box |
| **Year** | Year of publication | Text box |
| **Journal** | Journal abbreviation | Text box |
| **Country** | Country in which the study was conducted | Text box |
| **Type of Care** | Where does the study fall on the care continuum | DROP DOWN MENU (Acute Care, Long-term care, Rehabilitation, Community, Telehealth) |
| **Setting** | Setting (i.e., hospital, community, rehab) | Text box |
| **Study Design** | Design of study | DROP DOWN MENU (RCT, cluster-RCT) |
| **# of Arms** | Number of intervention arms | Text box |
| **Length of F/U** | Longest length of follow- up | Text box |
| **Population Age** | Population age | DROP DOWN MENU (65 and older, mixed age group [i.e., 40-70 years old]) |
| **# of patients** | Number of patients randomised | Text box |
| **Patient Type** | Description of patient group (i.e., dementia pts, hip fracture pts, healthy elderly, or other diagnostic categories/conditions) | Text box |
| **Inclusion criteria** | Description of inclusion criteria for recruitment | COMBINATION CELL (Text To Columns with a delimiter of comma) |
| **Exclusion criteria** | Description of exclusion criteria for recruitment | COMBINATION CELL (Text To Columns with a delimiter of comma) |
| **Comorbidities reported** | Comorbid or chronic conditions reported | DROP DOWN MENU (YES, NO) |
| **# of Comorbid/Chronic conditions** | Number of comorbid conditions reported | Text box |
| **List conditions** | List conditions separated by commas | COMBINATION CELL (Text To Columns with a delimiter of comma) |
| **Gender** | Gender reported | DROP DOWN MENU (YES, NO) |
| **Ethnicity** | Ethnicity reported | DROP DOWN MENU (YES, NO) |
| **# of ethnic categories** | Number of ethnic categories | Text box |
| **Living status at baseline** | Status at baseline reported | DROP DOWN MENU (YES, NO) |
| **CGA conducted** | Comprehensive Geriatric Assessment (CGA) performed | DROP DOWN MENU (YES, NO) |
| **# of CGA components** | Number of scales/domains assessed in CGA | Text box |
| **CGA Components** | List all the CGA components separated by commas | COMBINATION CELL (Text To Columns with a delimiter of comma) |
| **Standard of care defined?** | If standard of care is reported is a definition provided? | DROP DOWN MENU (YES, NO) |
| **Geriatrician involved?** | Geriatrician involved | DROP DOWN MENU (YES, NO) |
| **Role of geriatrician** | Role of geriatrician in the intervention | Text box |
| **# of team members present** | Number of team members involved in intervention | Text box |
| **List team** | List team members or disciplines separated by commas | COMBINATION CELL (Text To Columns with a delimiter of comma) |
| **# of outcomes reported** | Number of outcomes reported | Text box |
| **Cognitive functioning?** | Cognitive functioning reported | DROP DOWN MENU (YES, NO) |
| **Scale** | List the name of the scale | If multiple separate by commas |
| **Outcome reported as:** | How is the outcome reported | DROP DOWN MENU (Continuous, Dichotomous, Survival/Time to event, Costing, Other) |
| **Functional ability** | Functional ability reported | DROP DOWN MENU (YES, NO) |
| **Scale** | List the name of the scale | Text box |
| **Outcome reported as:** | How is the outcome reported | DROP DOWN MENU (Continuous, Dichotomous, Survival/Time to event, Costing, Other) |
| **Quality of Life** | Quality of life reported | DROP DOWN MENU (YES, NO) |
| **Scale** | List the name of the scale | Text box |
| **Outcome reported as:** | How is the outcome reported | DROP DOWN MENU (Continuous, Dichotomous, Survival/Time to event, Costing, Other) |
| **Caregiver burden** | Caregiver burden reported | DROP DOWN MENU (YES, NO) |
| **Scale** | List the name of the scale | Text box |
| **Outcome reported as:** | How is the outcome reported | DROP DOWN MENU (Continuous, Dichotomous, Survival/Time to event, Costing, Other) |
| **Falls** | Falls reported | DROP DOWN MENU (YES, NO) |
| **Fracture** | Fracture reported | Text box |
| **Type of fracture** | List types of fractures | DROP DOWN MENU (YES, NO) |
| **# of pts living at home** | Number of patients living at home reported | DROP DOWN MENU (YES, NO) |
| **# of pts in long-term care** | Number of patients in long- term care reported | DROP DOWN MENU (YES, NO) |
| **# of pts admitted to acute care** | Number of patients admitted to acute care reported | DROP DOWN MENU (YES, NO) |
| **# of pts admitted to long-term care** | Number of patients admitted to long-term care reported | DROP DOWN MENU (YES, NO) |
| **LOS** | Length of stay in the hospital | DROP DOWN MENU (YES, NO) |
| **# of outpatient visits** | Number of outpatient visits | DROP DOWN MENU (YES, NO) |
| **# of physician visits** | Number of trips to the doctor’s office (including physician visits to the home) | DROP DOWN MENU (YES, NO) |
| **# of ED visits** | Number of emergency department visits | DROP DOWN MENU (YES, NO) |
| **Mortality** | Number of patients who died (after randomization) | DROP DOWN MENU (YES, NO) |
| **Other** | List other outcomes | Text box |
| **Scale** | List scales | Text box |
| **Outcome reported as:** | How is the outcome reported | DROP DOWN MENU (Continuous, Dichotomous, Survival/Time to event, Costing, Other) |
