# Supplementary material for: An evaluation of the comparative effectiveness of geriatrician-led comprehensive geriatric assessment for improving patient and healthcare system outcomes for older adults: a protocol for a systematic review and network meta-analysis
Source: Syst Rev. 2017 Mar 24;6:65. doi: 10.1186/s13643-017-0460-4 (PMC5366126; doi:10.1186/s13643-017-0460-4)
Supplement: Supplementary file 5 — Authorization for funding. Funding agreement with the Canadian Institutes of Health Research (CIHR) and the Strategy for Patient Oriented Research (SPOR). (PDF 45 kb) [file 13643_2017_460_MOESM5_ESM.pdf]

AUTHORIZATION FOR FUNDING

CIHR (Canadian Institutes of Health Research) has approved funding as detailed below. Subject to the approbation of funding by Parliament, these funds will be made available to the business officer at the indicated institution for disbursement.

AUTORISATION DE FINANCEMENT

IRSC (Instituts de recherche en santé du Canada) vous accorde les fonds tel qu'indiqué ci-dessous. Suivant l'affectation des crédits par le Parlement du Canada, les fonds seront mis à la disposition du trésorier de l'établissement indiqué qui s'occupera des versements.

201607NKS-372688-NKS-CEAJ-24847

16/12/2016

Institution Paid/Établissement chargé d'administrer les fonds:
St. Michael's Hospital (Toronto)

Recipient(s)/Bénéficiaire(s): Straus, Sharon Elizabeth

Medicine
Faculty of Medicine
St. Michael's Hospital (Toronto)

Dr. Jayna Marie Holroyd-Leduc, Dr. Heather Anne Armson, Dr. Barbara Liu, Dr. Andrea Catherine Tricco, et al.

Program/Programme: Operating Grant: Pan-Canadian SPOR Network in Primary & Integrated Health Care Innovations – Knowledge Synthesis Grants
Grant New

Primary Institute/Institut principal: Aging

Project Title/Titre du projet:

Evaluating the comparative effectiveness of comprehensive geriatric assessment for improving patient and healthcare system outcomes: A systematic review and network meta-analysis

Co-investigator(s) & Associates/Supervisor(s)/Host/Co-chercheur(s)/Directeur(s) de recherche/Hôte:

Ms. Charlene Soobiah, Dr. Jennifer Ann Watt, Dr. Jemila Hamid, Dr. Sharon Amy Marr, et al.

PAYMENT DETAILS/DÉTAILS DES VERSEMENTS — Funding Reference Number/ No. de Référence du financement: NKS — 150583

Period<br>Période	Type	Amount by Type<br>Montant par type	Total by Fiscal Year<br>Total par exercice	
01/12/2016 to 31/03/2017	Operating	$24,986	$24,986	2016-17

Progress Report Required:<br>Rapport des progrès réalisés requis: **Not Applicable**	Application to Renew Funding Required:<br>Demande de renouvellement des fonds requis: **Non-Renewable**

NOTES:

The term of this grant is effective December 1, 2016 and expires on November 30, 2017. CIHR funds are conditional upon continued receipt of matching partner funds.
CIHR contribution: $24,986
Partner contributions:
• Regional Geriatric Program – Toronto: $10,000 (In-kind)
• Alberta Health Services Calgary Zone: $20,000 (In-kind)
• Regional Geriatric Program – Central: $12,000 (Cash) $10,000 (In-kind)
• Stonechurch Family Health Ctr- McMaster University: $5,000 (In-kind)
• University of Toronto - Dept of Geriatric Medicine: $11,000 (Cash)
• Saskatoon Health Region: $10,000 (In-kind)
The total value of this grant is $102,986. CIHR will administer its contribution, paid directly to the institution.

CIHR and any participating partners require that their contribution to your research project be acknowledged in all written and oral presentations of your research results, including scientific articles, news releases, news conferences, public lectures and media interviews. Please see CIHR's Guidelines on Public Communication which are enclosed for more information on public communication and acknowledgement requirements.

You received this funding because your colleagues volunteered their time to assist CIHR with the review of your application. We ask that, as a recipient of CIHR funding, you will participate in CIHR peer review activities if invited.

By drawing on the funds provided through this grant/award you agree to the terms and conditions set out in the attached "Conditions of Funding", any breach of which may result in CIHR taking remedial action as described therein.

If you are in receipt or become eligible to receive any funding from another source for any part of this project you must advise CIHR immediately by following the instructions outlined in the "Funding Overlap Declaration" form http://www.cihr-irsc.gc.ca/e/797.html. Failure to self-declare overlap could lead to CIHR cancelling all funding related to this grant.

CIHR will require you to submit an electronic Final Report through the Research Reporting System on ResearchNet for this grant. Instructions will be provided through an email notification from the ResearchNet system once the activity becomes available.
